# Hemoadsorption corrects hyperresistinemia and restores anti-bacterial neutrophil function

**DOI:** 10.1186/s40635-017-0150-5

**Published:** 2017-08-04

**Authors:** Anthony Bonavia, Lauren Miller, John A. Kellum, Kai Singbartl

**Affiliations:** 1 Department of Anesthesiology and Perioperative Medicine, Penn State Health, Hershey, PA USA; 2 Department of Critical Care Medicine, University of Pittsburgh, Pittsburgh, PA USA; 3 Center for Critical Care Nephrology, University of Pittsburgh, Pittsburgh, PA USA; 4 Department of Critical Care Medicine, Mayo Clinic, 5777 East Mayo Boulevard, Phoenix, AZ 85054 USA

**Keywords:** Resistin, Septic shock, Neutrophil dysfunction, Reactive oxygen species, Hemoadsorption therapy

## Abstract

**Background:**

Mounting evidence suggests that sepsis-induced morbidity and mortality are due to both immune activation and immunosuppression. Resistin is an inflammatory cytokine and uremic toxin. Septic hyperresistinemia (plasma resistin >20 ng/ml) has been associated with greater disease severity and worse outcomes, and it is further exacerbated by concomitant acute kidney injury (AKI). Septic hyperresistinemia disturbs actin polymerization in neutrophils leading to impaired neutrophil migration, a crucial first-line mechanism in host defense to bacterial infection. Our experimental objective was to study the effects of hyperresistinemia on other F-actin-dependent neutrophil defense mechanisms, in particular intracellular bacterial clearance and generation of reactive oxygen species (ROS). We also sought to examine the effects of hemoadsorption on hyperresistinemia and neutrophil dysfunction.

**Methods:**

Thirteen patients with septic shock and six control patients were analyzed for serum resistin levels and their effects on neutrophil migration. In vitro, following incubation with resistin-spiked serum samples, *Pseudomonas* *aeruginosa* clearance and ROS generation in neutrophils were measured. Phosphorylation of 3-phosphoinositide-dependent protein kinase-1 (PDPK1) was assessed using flow cytometry. In vitro hemoadsorption with both Amberchrome™ columns (AC) and CytoSorb® cartridges (CC) were used to test correction of hyperresistinemia. We further tested AC for their effect on cell migration and ROS generation and CC for their effect on bacterial clearance.

**Results:**

Patients with septic shock had higher serum resistin levels than control ICU patients and showed a strong, negative correlation between hyperresistinemia and neutrophil transwell migration (*ρ*= − 0.915, *p* < 0.001). In vitro, neutrophils exposed to hyperresistinemia exhibited twofold lower intracellular bacterial clearance rates compared to controls. Resistin impaired intracellular signaling and ROS production in a dose-dependent manner. Hemoadsorption with AC reduced serum concentrations of resistin and restored neutrophil migration and generation of ROS to normal levels. Hemoadsorption with CC also corrected hyperresistinemia and reconstituted normal intracellular bacterial clearance.

**Conclusions:**

Septic hyperresistinemia strongly correlates with inhibition of neutrophil migration in vitro. Hyperresistinemia itself reversibly impairs neutrophil intracellular bacterial clearance and ROS generation. Hemoadsorption therapy with a clinically approved device corrects hyperresistinemia and neutrophil dysfunction. It may therefore provide a therapeutic option to improve neutrophil function during septic hyperresistinemia and ultimately alleviate immunosuppression in this disease state.

## Background

Mounting evidence suggests that sepsis-induced morbidity and mortality are not only due to immune activation and inflammation but also due to immunosuppression [1, 2]. Persistent septic foci and increased burden with opportunistic microorganisms are hallmarks of sepsis-induced immunosuppression, consistent with an inability to clear primary infections and an increased risk for secondary infections [3, 4]. These problems extend well beyond the initial hospital admission, increasing morbidity and mortality rates in sepsis survivors for many years afterwards [5, 6].

Sepsis-induced immunosuppression affects all types of immune cells. Neutrophils are pivotal components of innate immunity and provide the first line of defense against invading microorganisms. After recruitment to the site of invading microorganisms, they phagocytose, kill, and digest the microorganisms via well-orchestrated processes involving reactive oxygen species (ROS). However, neutrophils in sepsis demonstrate an immunosuppressed phenotype [7]. Reduced bacterial clearance, diminished production of ROS, and impaired recruitment to the site of infection are the most prominent features [8, 9]. Available experimental data also reveal that neutrophil dysfunction develops prior to secondary infections and that patients with the most severely impaired neutrophil function carry the highest risk for secondary infections [9]. However, the mechanisms involved in these processes are still poorly understood.

Our group and others have shown that resistin, an inflammatory molecule and well-known uremic toxin, is elevated during septic shock, especially in patients with concomitant acute kidney injury (AKI) [10–13]. Resistin is also elevated in chronic kidney disease and diabetes mellitus [14–16]. However, serum resistin concentrations during sepsis/septic shock do not seem to be associated with underlying chronic kidney disease or diabetes mellitus [11]. Resistin is an early-phase 12.5 kDa cysteine-rich protein that contains a 17 amino acid signaling peptide [17]. It is known to be released by myeloid cells, particularly neutrophils, and its levels are positively correlated with disease severity (e.g., APACHE II scores) [11, 18]. Its physiologic role is uncertain, as is its cellular mechanism of action. There are no identified resistin receptors on neutrophils, nor are there any known resistin-dependent intracellular signaling pathways. Resistin concentrations ≤20 ng/ml do not appear to negatively affect neutrophil migration [10], whereas hyperresistinemia in sepsis has been associated with a greater disease severity and worse outcomes [11, 13, 19]. In some patients, resistin levels remain elevated for 4 weeks or longer after the initial presentation [12, 13].

Our prior work has demonstrated that hyperresistinemia (plasma resistin >20 ng/ml) in the setting of septic shock and AKI reversibly hinders neutrophil migration in vitro, likely by impairing F-actin formation [10]. However, standard renal replacement therapy (RRT) does not appear to correct hyperresistinemia and concomitant neutrophil dysfunction [10, 20]. Our current work revolves around the overall hypothesis that hyperresistinemia impairs bacterial clearance and inhibits intracellular generation of ROS both of which also depend on correct actin polymerization [21–24]. We further sought to examine the effects of hemoadsorption techniques on serum resistin levels as proof-of-concept for reversing resistin-induced neutrophil dysfunction [1, 2].

## Methods

### Reagents

All reagents were purchased from Sigma Aldrich unless stated otherwise.

### Human subjects

The University of Pittsburgh Institutional Review Board approved the study. After informed consent, blood was drawn from 13 patients with septic shock and from six post-operative ICU patients without signs of sepsis or AKI. Plasma samples were isolated and stored at −80 °C. Septic shock was defined according to the American College of Chest Physicians/Society of Critical Care Medicine consensus conference criteria [25]. AKI was defined according to the Risk, Injury, Failure, Loss, and End-stage Kidney (RIFLE) criteria; only patients with RIFLE-F were included [26]. We excluded patients who were pregnant, younger than 18 years, already receiving RRT, receiving immunosuppressive therapy, and with a history of hematologic malignancy or chronic kidney disease. Eight out of 13 patients with septic shock had no AKI, the remaining five patients with septic shock had RIFLE-F AKI [26]. In these patients, blood samples were collected before commencement of RRT.

### Plasma resistin concentrations

The concentrations of human resistin were measured using ELISA kits from R&D Systems (R&D Systems, Minneapolis, MN, USA).

### Neutrophilic differentiation of NB4 acute promyelocytic leukemia cells

We used neutrophilic-differentiated NB4 cells (NB4^PMN^) to study the effect of hyperresistinemia on neutrophil function in a standard and reproducible way. Neutrophilic differentiation was achieved by previously described methods [10]. Briefly, NB4 cells were treated with 1 μM all-trans retinoic acid (ATRA) for 6 days in culture media. Successful neutrophilic differentiation was assessed using CD11b, CD35, and CD71 surface markers (BD Biosciences) with flow cytometry. For some experiments, NB4^PMN^ were incubated for 1 h in human control serum (Atlanta Biologicals Flowery Branch, GA, USA) spiked with different concentrations of recombinant human resistin (R&D Systems, Minneapolis, MN, USA) at 20, 50, 100 ng/mL to replicate normal circulating vs. septic resistin concentrations.

### Transwell migration assays with NB4^PMN^ cells

As described previously, NB4^PMN^ cells were incubated in human control serum or patient samples for 1 h prior to transwell migration assay [10]. Five-micrometer transwell chambers (Corning Inc., Corning, NY, USA) coated with fibrinogen were used to evaluate chemotaxis. Migration without N-formylmethionyl-leucyl-phenylalanine (fMLP) or toward 100 nM fMLP was allowed for 2 h (37 °C, 5% carbon dioxide). Migrated cells in the bottom well were counted via flow cytometry. All samples were run in triplicates. Transwell migration of NB4^PMN^ cells was calculated as ratio of the cell concentration in the lower chamber and the concentration of cells seeded in the upper chamber prior to migration (C6 flow cytometer; BD Biosciences, San Jose, CA). The final results are presented relative to control samples to normalize for inter-assay variability.

### Reactive oxygen species generation and quantification

Reactive oxygen species (ROS) generation was measured using CellROX Deep Red Reagent probe (Invitrogen, Carlsbad, CA, USA). 1 × 10^6^ cells/mL NB4^PMN^ cells were suspended and washed with 1% BSA in PBS and stimulated without or with 100 ng/mL PMA for 10 min at 37 °C. Ice cold PBS was added to stop the reaction, and diluted CellROX probe (concentration 7.5 μM) was added and incubated for 10 min at 37 °C. Samples were fixed in 4% formaldehyde in PBS (Boston BioProducts, Boston, MA, USA) and analyzed by flow cytometry. The final results are presented relative to control to normalize for inter-assay variability.

### NB4^PMN^ phosflow intracellular staining of PDPK1

NB4^PMN^ cells were incubated in human serum spiked with 20, 50, and 100 ng/mL human recombinant resistin for 1 h and then subjected to a modified assay adapted from BD Phosflow Protocols for Human PBMCs. Briefly, NB4^PMN^ cells were stimulated for 1 min at 37 °C with or without 1 μM fMLP. Cells were immediately fixed with 4% formaldehyde in PBS and permeabilized on ice with BD Phosflow Perm Buffer III (BD Biosciences, San Jose, CA, USA). Alexa Fluor 647 Mouse anti-PDPK1 (pS241) (BD Biosciences, San Jose, CA, USA) was added to all samples. After 1-h incubation at room temperature in the dark, samples were analyzed by flow cytometry. Results are displayed as median fluorescence intensity (MFI) relative to control samples.

### NB4^PMN^ phagocytosis and bacterial clearance assay

We used *Pseudomonas aeruginosa* strain UI-18 (PA-7) (ATCC, Manassas VA, USA) to examine the phagocytic and bacterial clearance capacity of NB4^PMN^ cells according to previously described methods [27]. Briefly, bacteria were suspended at 10^7^ cells/mL in PBS and opsonized with 10% human serum with end-over-end rotation at 37 °C. Cytochalasin B (10 μM) served as positive control for actin cytoskeleton inhibition. 10^7^ NB4^PMN^ cells with or without cytochalasin B were added to opsonized bacteria with end-over-end rotation at 37 °C. One milliliter of samples were taken after 30 min from the experimental tubes, added to ice cold PBS and centrifuged. Supernatants yielding extracellular bacteria were collected, diluted according to protocol instructions, and plated on *P. aeruginosa* isolation agar (BD Diagnostics Sparks, MD, USA). One milliliter of PBS with 0.05% saponin was added to the neutrophil pellet. Cells were disrupted using a glass homogenizer and plated. Extracellular bacteria suspensions and intracellular suspension plates were incubated at 37 °C overnight. Colonies were counted the following day. The results are displayed as colony forming units (CFU) relative to control.

### In vitro removal of resistin by resin adsorption therapy

We used the Amberchrome CG161M™ resin (Rohm and Haas Company, Philadelphia, PA, USA) for cytokine removal as described by Cataluppi et al. [28]. Recombinant resistin and serum lots were identical for all experiments. Briefly, resin was activated with 50% methanol and extensively washed with PBS before use. One inch inner diameter chromatography columns (Bio-Rad, Hercules, CA, USA) were treated with Sigmacote™ and washed with distilled water before use. Primed chromatography columns were packed with 2 mL of activated resin and circulated human serum without or with 100 ng/mL recombinant resistin at a flow rate of 0.3 mL/min using a peristaltic pump. Samples were removed from the reservoir at 60, 120, and 180 min. Serum samples before and after resin adsorption therapy (RAT) were kept at −80 °C for ROS testing and transwell migration assay. Other neutrophil function assays were not possible due to sample volume limitations.

### In vitro removal of resistin by hemoadsorption

Hemoadsorption cartridges (CytoSorb®) were provided by Cytosorbents Corporation (Monmouth Junction, NJ, USA). Removal of resistin was characterized by spiking recombinant human resistin into 10 mL of normal human serum. Provided columns were primed with 30 mL PBS and subjected to a peristaltic pump circuit at a flow rate of 0.8 mL/min at 37 °C to mimic clinical conditions. Samples were taken at 30, 60, 120, and 180 min. Serum samples were also taken before and after cartridge treatment and kept at −80 °C for bacterial clearance assay. Other neutrophil function assays were not possible due to sample volume limitations.

### Statistics

All data are presented as median (interquartile range). Statistical analysis included, where indicated, test for normality (Shapiro-Wilks), paired and unpaired *t* tests, one-way analysis of variance (ANOVA) with Tukey multiple comparisons test, two-way ANOVA with Tukey multiple comparisons test, Wilcoxon matched-pairs signed-rank test, Spearman rank correlation, and Mann-Whitney test. A *p* value of less than 0.05 was considered statistically significant.

## Results

**Fig. 1:**
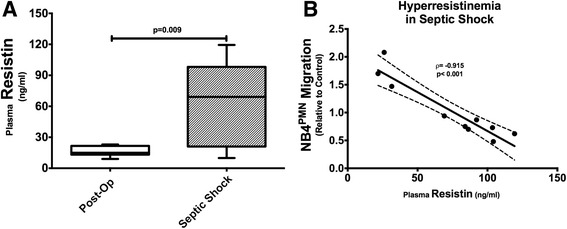
**a** Patients with septic shock had higher plasma resistin levels compared to postoperative ICU patients without sepsis or AKI. **b** There is a strong correlation between hyperresistinemia and inhibition of transwell migration in NB4^PMN^ cells suspended in serum from patients with septic shock

In normal control subjects, plasma resistin concentrations are usually <20 ng/mL [16, 20]. However, patients with septic shock show drastically elevated plasma resistin concentrations when compared to postoperative critically ill patients without sepsis or AKI (Fig. 1a). The correlation between plasma resistin concentration and inhibition of NB4^PMN^ transwell migration in our overall cohort of ICU patients is very poor and statistically not significant (*ρ*= − 0.375, *p* = 0.113). However, there is a very strong, direct correlation between plasma resistin concentrations and inhibition of NB4^PMN^ transwell migration in patients with septic shock and hyperresistinemia, i.e., plasma resistin concentrations >20 ng/mL (Fig. 1b).

**Fig. 2:**
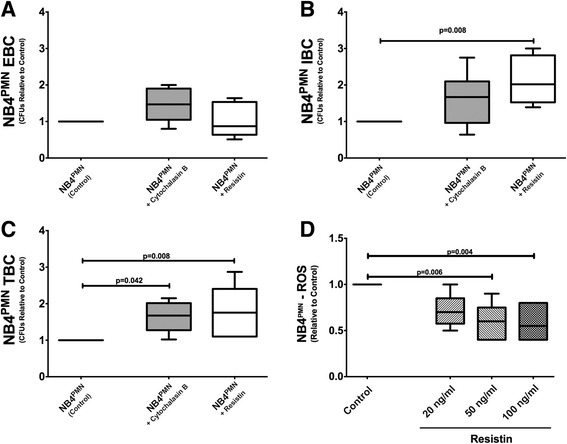
Neither cytochalasin B nor resistin affects extracellular bacterial clearance by NB4^PMN^ cells (**a**). NB4^PMN^ cells exposed to resistin (100 ng/ml) exhibited lower intracellular bacterial clearance rates compared to controls but similar to that observed with cytochalasin B incubation (**b**). Total bacterial clearance was significantly impaired by both cytochalasin B and resistin (100 ng/ml) (**c**). Resistin impairs reactive oxygen species production by neutrophils in a dose-dependent manner (**d**). *N* = 6 for all experiments

Resistin impairs intracellular actin polymerization, which is a crucial process for many neutrophil functions, including effective neutrophil migration [10]. We therefore studied the effect of resistin on key antibacterial mechanisms that are also F-actin-dependent. Using cytochalasin B as a positive control for inhibition of actin polymerization, our experiments show that neither cytochalasin B nor resistin do affect extracellular clearance of *P. aeruginosa* (Fig. 2a). However, resistin significantly inhibits intracellular bacterial clearance (Fig. 2b), a process that depends on generation of ROS. Resistin consequently decreases total bacterial clearance similar to that seen after pre-treatment with cytochalasin B (Fig. 2c). Resistin further diminishes the generation of ROS in a dose dependent manner (Fig. 2d), providing a possible mechanism for our observed reduction in intracellular bacterial clearance.

**Fig. 3:**
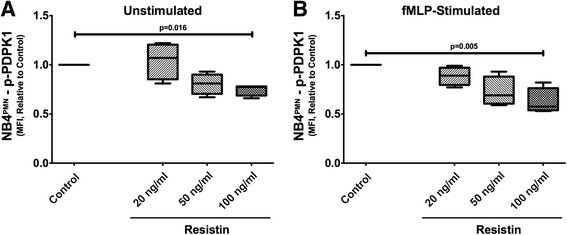
Resistin impairs PDPK1 phosphorylation in a dose-dependent manner (**a**). This effect appears more pronounced after neutrophil stimulation with fMLP (**b**). *N* = 4 for all experiments

While no resistin receptor has been identified in humans, resistin is known to interfere with intracellular signaling involving the phosphatidylinositol 3-kinase (PI3K) pathway which is crucial for key neutrophil functions, including migration and generation of ROS [14]. Our in vitro incubation experiments show that increasing concentrations of resistin appear to inhibit PDPK1 phosphorylation in a dose-dependent fashion (Fig. 3a, b). Phosphorylation of PDPK1 is a pivotal, upstream event within the PI3K pathway, as it regulates each step of cell migration. Resistin affects both constitutive (Fig. 3a) and stimulation-dependent phosphorylation of PDPK1 (Fig. 3b).

**Fig. 4:**
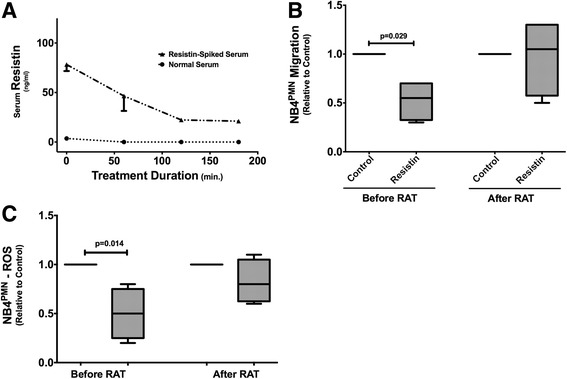
Hemoadsorption therapy using Amberchrome CG161M™ columns reduces serum concentrations of resistin (**a**). Amberchrome CG161M™ treatment als restores impaired NB4^PMN^ cell transwell migration (**b**) and ROS production back to normal levels (**c**). *N* = 4 for all experiments

As conventional RRT does not appear to be sufficient to correct hyperresistinemia [10, 20], we sought to test other extracorporeal blood purification techniques regarding their ability to clear elevated resistin levels. In vitro circulation experiments with resistin-spiked serum using Amberchrome CG161M™ reveal a more than 50% reduction in serum resistin levels (Fig. 4a). This degree of reduction leads to a significant improvement in both NB4^PMN^ transwell migration (Fig. 4b) and generation of ROS (Fig. 4c). Here, incubation of NB4^PMN^ cells in resistin-spiked serum after Amberchrome CG161M™ treatment restores both transwell migration and generation of ROS back to control levels.

**Fig. 5:**
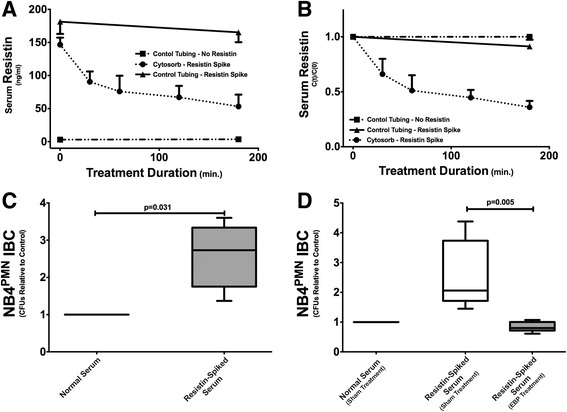
Hemoadsorption treatment with Cytosorb™ corrects hyperresistinemia by removing more than 50% of excessive resistin from resistin-spiked serum (**a**, **b**). Incubation of NB4^PMN^ cells in resistin-spiked serum prior to hemoadsorption treatment reveals impaired intracellular *P. aeruginosa* clearance compared to incubation in control serum (**c**). However, incubation of NB4^PMN^ cells in resistin-spiked serum after hemoadsorption treatment shows restitution of normal intracellular bacterial clearance (**d**)

In vitro treatment of resistin-spiked serum with the clinically approved hemoadsorption device Cytosorb® also corrects hyperresistinemia and restores neutrophil function. A 3-h treatment with Cytosorb® in a clinically scaled circuit reveals an approximately 50% reduction in serum resistin levels (Fig. 5a, b). This amount of reduction translates into a normalization of intracellular bacterial clearance in NB4^PMN^ cells incubated with Cytosorb™-treated serum (Fig. 5d). Sham treatment, i.e., no Cytosorb® cartridges in the circuit, does not correct impaired intracellular bacterial clearance (Fig. 5d). Here, the number of CFU is similar to those seen with resistin-spiked serum prior to treatment (Fig. 5c).

## Discussion

Immunosuppression has emerged as a key factor in sepsis-related morbidity and mortality [1, 2]. The development of AKI during sepsis further increases morbidity and mortality in these patients [29]. Resistin is an inflammatory cytokine and uremic toxin that is associated with worse outcomes from sepsis and septic shock. The exact biological effects of resistin and the underlying intracellular mechanisms remain unknown. Moreover, conventional renal replacement therapies do not appear to be able to clear excessive amounts of resistin [10, 20]. Here, we present a translational study to further delineate the relationship between excessive amounts of resistin, i.e., hyperresistinemia, and key neutrophil functions. We further shed light on the intracellular effects of resistin and assess the role of hemoadsorption as a treatment option for hyperresistinemia.

Using a cohort of patients with septic shock and control ICU patients, i.e., patients without AKI or septic shock, we were able to show a strong correlation between hyperresistinemia and impaired NB4^PMN^ transwell migration. Our findings are in agreement with studies in patients with end-stage renal disease showing impaired neutrophil chemotaxis in the setting of hyperresistinemia [14]. The observed strong correlation between plasma resistin concentration and inhibition of migration delivers further evidence for a dose-dependent effect of resistin on neutrophil function.

Resistin impairs intracellular actin polymerization, a key prerequisite for many neutrophil functions [30, 31]. We therefore studied the effect of resistin on other F-actin dependent neutrophil functions that are also crucial for antibacterial host defense [21, 22]. Whereas resistin did not appear to interfere with extracellular bacterial clearance, it clearly inhibited intracellular clearance of *P. aeruginosa* by NB4^PMN^. As resistin fails to affect extracellular bacterial clearance, we can postulate that resistin does not interfere with bacterial phagocytosis or extracellular clearance via neutrophil extracellular traps (NET), both of which are also crucial processes for neutrophil-mediated host defense [32]. The effects of resistin pre-treatment did not exactly match those observed with cytochalasin B pre-treatment. One can therefore hypothesize that mechanisms other than inhibition of actin polymerization also play a role. Nonetheless, inhibition of actin polymerization still appears to be a crucial mechanism in resistin-mediated neutrophil dysfunction.

Resistin also inhibited generation of ROS by NB4^PMN^ cells. Generation of ROS is one of the key mechanisms for intracellular clearance of bacteria in neutrophils. It also represents one of the final steps in a complex cascade of events necessary for effective antibacterial host defense. Our current work together with our previous findings [10, 33, 34] strengthen the hypothesis that hyperresistinemia, as it occurs during sepsis and AKI, negatively affects both neutrophil migration toward and clearance of bacteria. Here, impaired F-actin formation in neutrophils/NB4^PMN^ has emerged as the common intracellular hallmark of hyperresistinemia. Destabilization/disruption of actin polymerization negatively affects both correct cell migration and ROS formation.

We have previously shown impaired phosphorylation within the phosphoinositide 3-kinase (PI3K) pathway in neutrophils during AKI, in particular impaired phosphorylation of Akt [33]. The PI3K pathway is vital for proper actin polymerization in many different cell types [35]. Phosphoinositide-dependent kinase 1 (PDPK1) is considered a pivotal regulator within the PI3K pathway [36]. Our findings indicate that hyperresistinemia is able to decrease the constitutive phosphorylation of PDPK1 in unstimulated cells in a dose-dependent manner, although this effect is more pronounced in fMLP-stimulated cells.

Neutrophil chemotaxis is triggered by ligand-receptor binding and activation of PI3K classes 1a and 1b, which cause phosphatidylinositol triphosphate (PIP3) to accumulate at the leading edge of the migrating cell [37, 38]. PDPK1 is a constitutively active PI3K-dependent effector which plays an important role in the PI3K/Akt signaling pathway [36, 39]. Decreased activity of PDPK1 attenuates neutrophil chemotaxis in response to fMLP [40, 41]. Our study provides evidence that the inhibitory effects of resistin are PDPK1 mediated. The effect of resistin on intracellular, but not extracellular bacterial clearance, argues against an effect on phagocytosis or NET formation, although previous studies have shown that PDPK1 regulates phagocytosis through multiple downstream enzymes of PDPK1 [42]. Akt (specifically the Akt2 isoform) is central to neutrophil function, with Akt2^−/−^ knockout mice exhibiting decreased cell migration, granule enzyme release, and superoxide radical production [43]. Further studies are needed to investigate if (and how) PDPK1 specifically affects Akt phosphorylation during hyperresistinemia.

The highest resistin levels are found in patients with septic shock and AKI (90–120 ng/ml), raising the possibility that elimination of this cytokine depends on glomerular function [10, 44–46]. There is a paucity of data regarding the clearance of resistin with conventional RRT. However, our studies support previous reports describing very limited resistin clearance with current modes of RRT [10, 20]. Hyperresistinemia is therefore likely to persist until either the production of resistin decreases or renal function improves, since there is currently no available treatment or intervention to correct hyperresistinemia.

Hemoadsorption therapy with a clinically approved device (CytoSorb™) removes cytokines (interleukin-6, interleukin-10, and tumor necrosis factor, amongst others) and improves short-term survival in a rat model of endotoxemia [47, 48] [1, 3, 4]. Case studies in patients with septic shock have corroborated these findings [49]. Moreover, recent studies indicate a role for hemoadsorption therapy that go beyond removal of common sepsis mediators [50]. In particular, improvement in neutrophil recruitment and bacterial clearance as well as reduced remote organ damage, e.g., secondary acute respiratory distress syndrome, all appear to contribute to improved survival following treatment with CytoSorb™ cartridges during experimental sepsis [51]. Treatment effects of hemoadsorption with CytoSorb™ cartridges are therefore consistent with a growing body of evidence that supports the concept of immune system incompetence as a driving factor in sepsis morbidity and mortality [1, 52].

We here utilized an in vitro model of serum adsorption based on that described by Cantaluppi et al. to remove resistin from serum samples with elevated levels of this cytokine [28]. Resin adsorption therapy with Amberchrome GC160M™ successfully removed resistin from resistin-spiked serum samples and restored both impaired neutrophil migration and ROS generation to normal levels. Similar results were obtained using CytoSorb™ cartridges. Treatment with CytoSorb™ cartridges lowered serum resistin concentration by approximately 50% and restored normal bacterial clearance in NB4^PMN^ cells.

Our study is the first, to our knowledge, to report effective removal of resistin from serum samples in this way and correction of resistin-induced cell dysfunction. Furthermore, we have illustrated the pharmacokinetics of resistin binding to lay the groundwork for future studies. The prospect is that extracorporeal apheresis treatment can reduce mortality and morbidity due to hyperresistinemia during sepsis by restoring immune competency, in particular neutrophil function.

Despite our exciting and sound findings, our study has limitations that require attention. First and foremost, our findings are the results of in vitro experiments that warrant further in vivo validation to corroborate these encouraging in vitro studies. Future studies have to show that (a) hyperresistinemia during sepsis negatively affects morbidity and mortality and (b) that correcting hyperresistinemia improves patient outcomes. Secondly, we utilized a differentiated neutrophil cell line for experimental work as opposed to primary neutrophils from hyperresistinemic patients. This was done for practical purposes, namely due to the large volume of blood required to extract a sufficient number of primary neutrophils as well as autologous plasma to perform the aforementioned experiments. It is our anticipation that once we develop a more complete and satisfactory mechanistic explanation for hyperresistinemic neutrophil dysfunction, we can replicate them in primary neutrophils from clinical samples. From a potentially therapeutic perspective, we would be interested in whether hemoadsorption of resistin can rescue primary neutrophil function in septic shock patients. If it cannot, then it would suggest that hemoadsorption may be effective only during the earliest stages of the disease.

## Conclusions

Hyperresistinemia in septic shock and AKI is believed to contribute to neutrophil dysfunction and subsequent immunosuppression. Our study has shed light on a possible mechanism of impaired intracellular bacterial clearance. Extracorporeal blood purification with resistin adsorption reverses hyperresistinemia-induced neutrophil dysfunction. In particular, hemoadsorption therapy with a clinically approved device reverses impaired bacterial clearance and may decrease infection rates in this vulnerable patient population. Further in vivo studies are needed to assess the feasibility of this therapeutic approach.

## Abbreviations

AC: Amberchrome CG161M™ resin
AKI: Acute kidney injury
CC: CytoSorb™ cartridges
fMLP: N-formylmethionyl-leucyl-phenylalanine
NB4^PMN^: Neutrophilic-differentiated NB4 cells
PDPK1: Phosphoinositide-dependent kinase-1
PI3K: Phosphatidylinositol-3-kinase
ROS: Reactive oxygen species
RRT: Renal replacement therapy
